# Serum Creatine, Not Neurofilament Light, Is Elevated in CHCHD10-Linked Spinal Muscular Atrophy

**DOI:** 10.3389/fneur.2022.793937

**Published:** 2022-02-17

**Authors:** Julius Järvilehto, Sandra Harjuhaahto, Edouard Palu, Mari Auranen, Jouni Kvist, Henrik Zetterberg, Johanna Koskivuori, Marko Lehtonen, Anna Maija Saukkonen, Manu Jokela, Emil Ylikallio, Henna Tyynismaa

**Affiliations:** ^1^Stem Cells and Metabolism Research Program, Faculty of Medicine, University of Helsinki, Helsinki, Finland; ^2^Clinical Neurosciences, Neurology, Helsinki University Hospital, Helsinki, Finland; ^3^Clinical Neurochemistry Laboratory, Sahlgrenska University Hospital, Mölndal, Sweden; ^4^Department of Psychiatry and Neurochemistry, Institute of Neuroscience and Physiology, The Sahlgrenska Academy at the University of Gothenburg, Mölndal, Sweden; ^5^Department of Neurodegenerative Disease, UCL Institute of Neurology, London, United Kingdom; ^6^UK Dementia Research Institute at UCL, London, United Kingdom; ^7^School of Pharmacy, University of Eastern Finland, Kuopio, Finland; ^8^Department of Neurology, Central Hospital of Northern Karelia, Joensuu, Finland; ^9^Division of Clinical Neurosciences, Turku University Hospital and University of Turku, Turku, Finland; ^10^Department of Neurology, Neuromuscular Research Center, Tampere University Hospital and Tampere University, Tampere, Finland; ^11^Neuroscience Center, Helsinki Institute of Life Science, University of Helsinki, Helsinki, Finland; ^12^Department of Medical and Clinical Genetics, University of Helsinki, Helsinki, Finland

**Keywords:** biomarker, NEFL, CHCHD10, SMAJ, creatine

## Abstract

**Objective:**

To characterize serum biomarkers in mitochondrial CHCHD10-linked spinal muscular atrophy Jokela (SMAJ) type for disease monitoring and for the understanding of pathogenic mechanisms.

**Methods:**

We collected serum samples from a cohort of 49 patients with SMAJ, all carriers of the heterozygous c.197G>T p.G66V variant in *CHCHD10*. As controls, we used age- and sex-matched serum samples obtained from Helsinki Biobank. Creatine kinase and creatinine were measured by standard methods. Neurofilament light (NfL) and glial fibrillary acidic protein (GFAP) were measured with single molecule array (Simoa), fibroblast growth factor 21 (FGF-21), and growth differentiation factor 15 (GDF-15) with an enzyme-linked immunosorbent assay. For non-targeted plasma metabolite profiling, samples were analyzed with liquid chromatography high-resolution mass spectrometry. Disease severity was evaluated retrospectively by calculating a symptom-based score.

**Results:**

Axon degeneration marker, NfL, was unexpectedly not altered in the serum of patients with SMAJ, whereas astrocytic activation marker, GFAP, was slightly decreased. Creatine kinase was elevated in most patients, particularly men. We identified six metabolites that were significantly altered in serum of patients with SMAJ in comparison to controls: increased creatine and pyruvate, and decreased creatinine, taurine, N-acetyl-carnosine, and succinate. Creatine correlated with disease severity. Altered pyruvate and succinate indicated a metabolic response to mitochondrial dysfunction; however, lactate or mitochondrial myopathy markers FGF-21 or GDF-15 was not changed.

**Conclusions:**

Biomarkers of muscle mass and damage are altered in SMAJ serum, indicating a role for skeletal muscle in disease pathogenesis in addition to neurogenic damage. Despite the minimal mitochondrial pathology in skeletal muscle, signs of a metabolic shift can be detected.

## Introduction

Spinal muscular atrophy Jokela type (SMAJ, MIM #615048) is a progressive adult-onset lower motor neuron disease (1). Muscle cramps, lower limb predominant muscle weakness, hyporeflexia, and progressive walking difficulties are typical symptoms (2). Disease onset is usually around 30–40 years, and EMG shows neurogenic alterations (1, 3). Muscle biopsies also show characteristic neurogenic findings, whereas only some patients have mild signs of mitochondrial myopathy (3). Some patients have received amyotrophic lateral sclerosis (ALS) or Charcot-Marie-Tooth (CMT) neuropathy as initial diagnoses (4–6), but a gene test of a single heterozygous variant, c.197G>Tp.G66V, in *coiled-coil-helix-coiled-coil-helix domain containing 10* (*CHCHD10*), sets the diagnosis of SMAJ (2). Importantly, more severe *CHCHD10* variants cause ALS and frontotemporal dementia (FTD) (5) or mitochondrial myopathy (7).

The CHCHD10 is a mitochondrial intermembrane space protein of unknown exact function, and both its absence and dominant pathogenic mutations affect mitochondrial respiration (8–13). Recent *Chchd10* mutant mice, with the ALS variant p.S59L, have displayed neuromuscular junction (NMJ) and motor neuron degeneration (9, 14). Induction of integrated stress response (ISR) has been reported both in the mice (8) and in fibroblasts of patients with ALS with the same mutation (14). However, studies of systemic consequences of CHCHD10-associated diseases in human patients have been rare.

Here, we present an investigation of blood metabolites and biomarkers in SMAJ. These results advance the understanding of the pathogenesis of CHCHD10-linked SMAJ disease.

## Patients and Methods

This study was approved by the medical ethics committee of Helsinki University Hospital. The participants gave written informed consent. In total, 49 individuals who were symptomatic verified carriers of CHCHD10 p.G66V were recruited (Table 1). Fasting serum samples were collected and stored at −80°C until the analysis. As on-site visits were not possible for the purpose of research, we used a questionnaire and a review of medical charts to collect clinical data. As an estimation of disease severity, we used a symptom-based score, which we modified from the motor and sensory defect part of a more comprehensive CMT neuropathy score (15) Supplementary Table 1). As controls, we used anonymized samples from Helsinki Biobank, which were requested to match the sex and age distributions of the study population, but with the exclusion of neurological disease diagnosis (ICD-10 code starting with G).

**Table 1:** Demographics of the study groups in different analyses (S = serum, P = plasma).

**Analysis**	**Patients**		**Controls**	
	***n* (M/F)**	**Age median (range)**	***n* (M/F)**	**Age median (range)**
S-NFL S-GFAP	49 (20/29)	65 (39–86)	45 (17/28)	64 (40–86)
S-FGF-21 S-GDF-15	49 (20/29)	65 (39–86)	24 (10/14)	63 (47–85)
P-Metabolite profiling	43 (19/24)	65 (39–86)	48 (18/30)	65 (34–86)
S-CK S-Creatinine	49 (20/29)	65 (39–86)	- (reference values)	- (reference values)
Symptom score	43 (16/27)	65 (39–85)	-	-

### Serum Measurements

Serum neurofilament light (NfL) and Glial fibrillary acidic protein (GFAP) concentrations were measured with single molecule array (Simoa) on an HD-X Analyzer using NF-light Advantage and GFAP Discovery kits (Quanterix, MA, USA). Serum fibroblast growth factor 21 (FGF-21) and growth/differentiation factor 15 (GDF-15) levels were measured with R&D Systems Quantikine ELISA kits (DF2100 and DGD150, MN, USA). Creatinine and creatine kinase (CK) was measured in an accredited laboratory, HUSLAB, Helsinki, Finland.

### Non-targeted Metabolite Profiling

Non-targeted metabolite profiling of plasma samples was performed using ultra-high performance liquid chromatography coupled to high-resolution mass spectrometry. Samples were randomized and deproteinized with acetonitrile. A small volume of each sample was pooled for the quality control sample and was analyzed at the beginning and after every 12th sample of the instrument worklist. To meet the wide diversity of sample components, all samples were analyzed using two different chromatographic techniques; i.e., reversed-phase (RP) and hydrophilic interaction chromatography (HILIC). In addition, data were acquired in both mass spectrometric electrospray ionization polarities; i.e., ESI+ and ESI– (16).

## Results

### NfL Is Not Elevated in SMAJ

**Figure 1:**
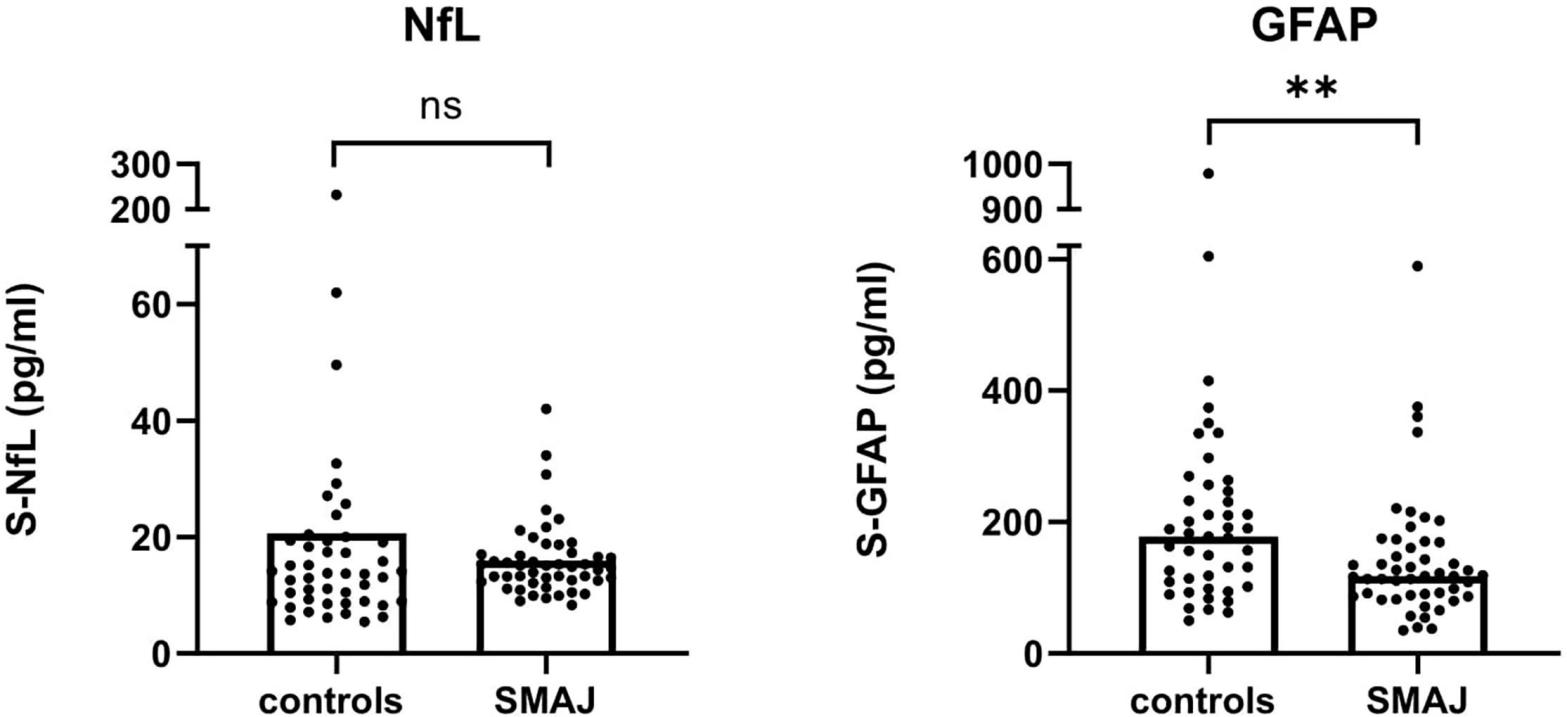
Serum neurofilament light is not elevated in SMAJ. Comparison of serum concentrations of NfL and GFAP between patients with SMAJ (*n* = 49) and controls (*n* = 45). The median per group is indicated by a bar. **p < 0.01, Mann–Whitney *U*-test.

The axonal injury protein NfL has shown to be a potential biomarker in a number of neurological conditions (17), including ALS, CMT, and SMA (18–20). However, the level of serum NfL was not significantly different in samples of a patient with SMAJ (median, 14.9 pg/ml), compared to controls (13.6 pg/ml) (Figure 1). A centrally expressed marker of astrogliosis (21), GFAP, was also not elevated in SMAJ; in contrast, a decreased level was observed (median, 118 pg/ml) as compared to controls (177 pg/ml) (*p* = 0.006, Figure 1).

### Altered Creatine Metabolism in SMAJ

**Figure 2:**
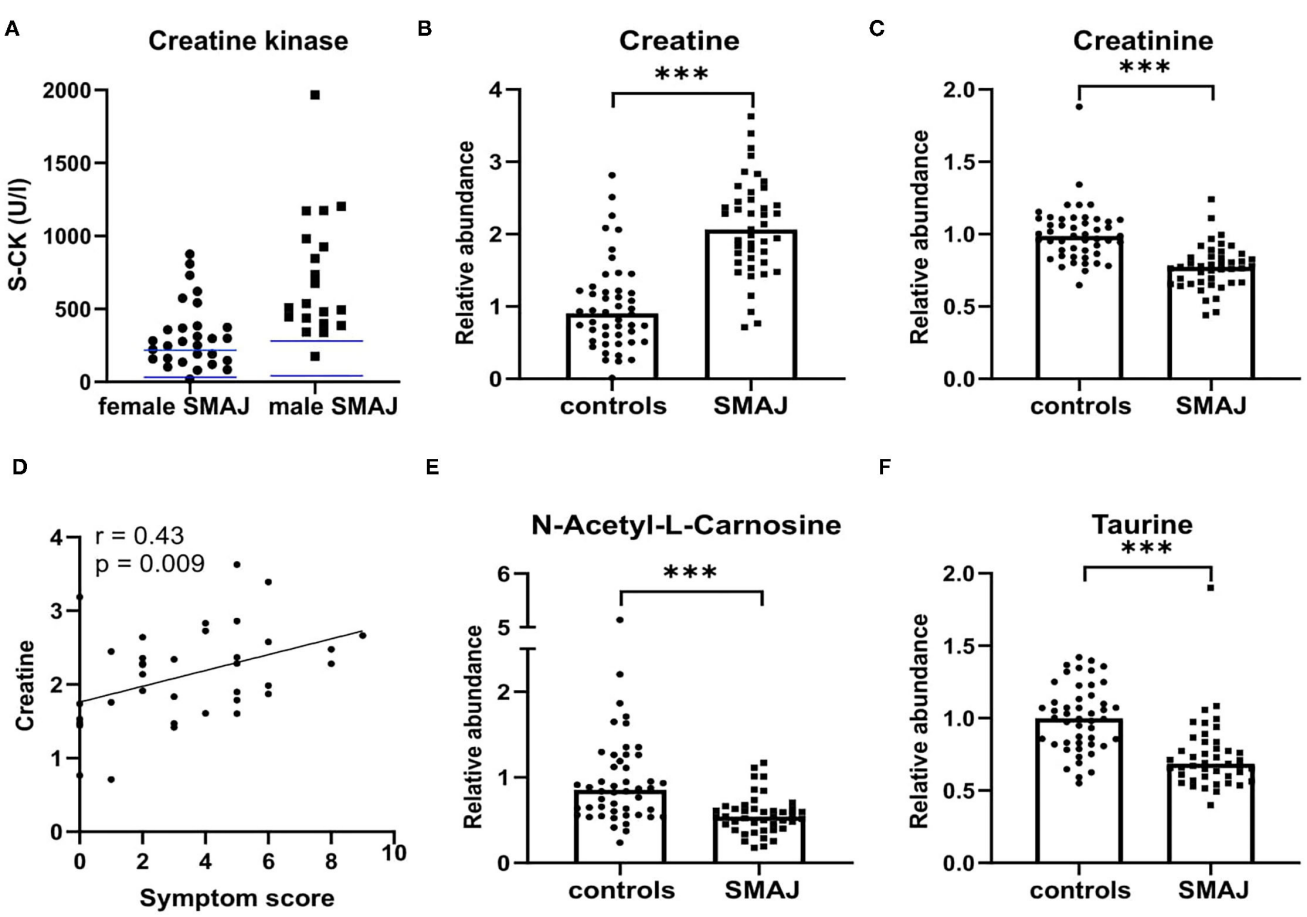
Biomarkers of muscle damage and mass in SMAJ. **(A)** Creatine kinase (S-CK) units of enzyme activity per liter of serum in female (*n* = 29) and male (*n* = 20) patients with SMAJ. Blue lines indicate the upper and lower age- and sex-matched laboratory reference values. Comparison of **(B)** creatine and **(C)** creatinine values between patients with SMAJ (*n* = 43) and controls (*n* = 48) measured with a mass spectrometer. Correlation of **(D)** creatine to a symptom score by Spearman correlation test *n* = 36. Comparison of **(E)** N-acetyl-L-carnosine and **(F)** taurine values between patients with SMAJ (*n* = 43) and controls (*n* = 48) measured with a mass spectrometer. For comparisons, median per group is indicated by a bar. ***p < 0.0001, Mann–Whitney *U*-test.

Previous studies indicated a CK elevation in SMAJ (1, 2). In this cohort, 60% of female patients with SMAJ had a CK above reference (mean, 318 U/l; reference range, 35–210 U/l), whereas 95% of male patients with SMAJ had an elevated CK (mean, 711 U/l; reference range, 40–280 U/l) (Figure 2A).

Next, we performed a non-targeted metabolite screen to identify the metabolites that could be used as markers of SMAJ pathogenesis. Six metabolites were significantly altered in SMAJ samples in comparison to controls: creatine (Fold Change, 2.09; adjusted *p*-value, 6.13 E-07), creatinine (FC, −1.30, adj.p 1.20 E-07), taurine (FC, −1.36, adj.p 1.08 E-06), N-acetyl-L-carnosine (FC, −1.77, adj.p 8.20 E-05), pyruvate (FC, 1.60; adj.p 0.00337), and succinate (FC, −1.37; adj.p 0.0127).

Creatine was increased in SMAJ (Figure 2B), whereas its secreted breakdown product, creatinine, was decreased (Figure 2C). We validated the metabolite profiling result by measuring creatinine levels in an accredited laboratory and found that the two measurements correlated well (*r* = 0.74, *p* < 0.001). Creatine was the only metabolite that correlated with the symptom score (*r* = 0.43, *p* = 0.009) (Figure 2D, Supplementary Table 2). Levels of N-acetyl-L-carnosine (Figure 2E) and taurine were also reduced in samples of patients with SMAJ (Figure 2F).

### Integrated Stress Response Markers Are Not Elevated in SMAJ Serum

**Figure 3:**
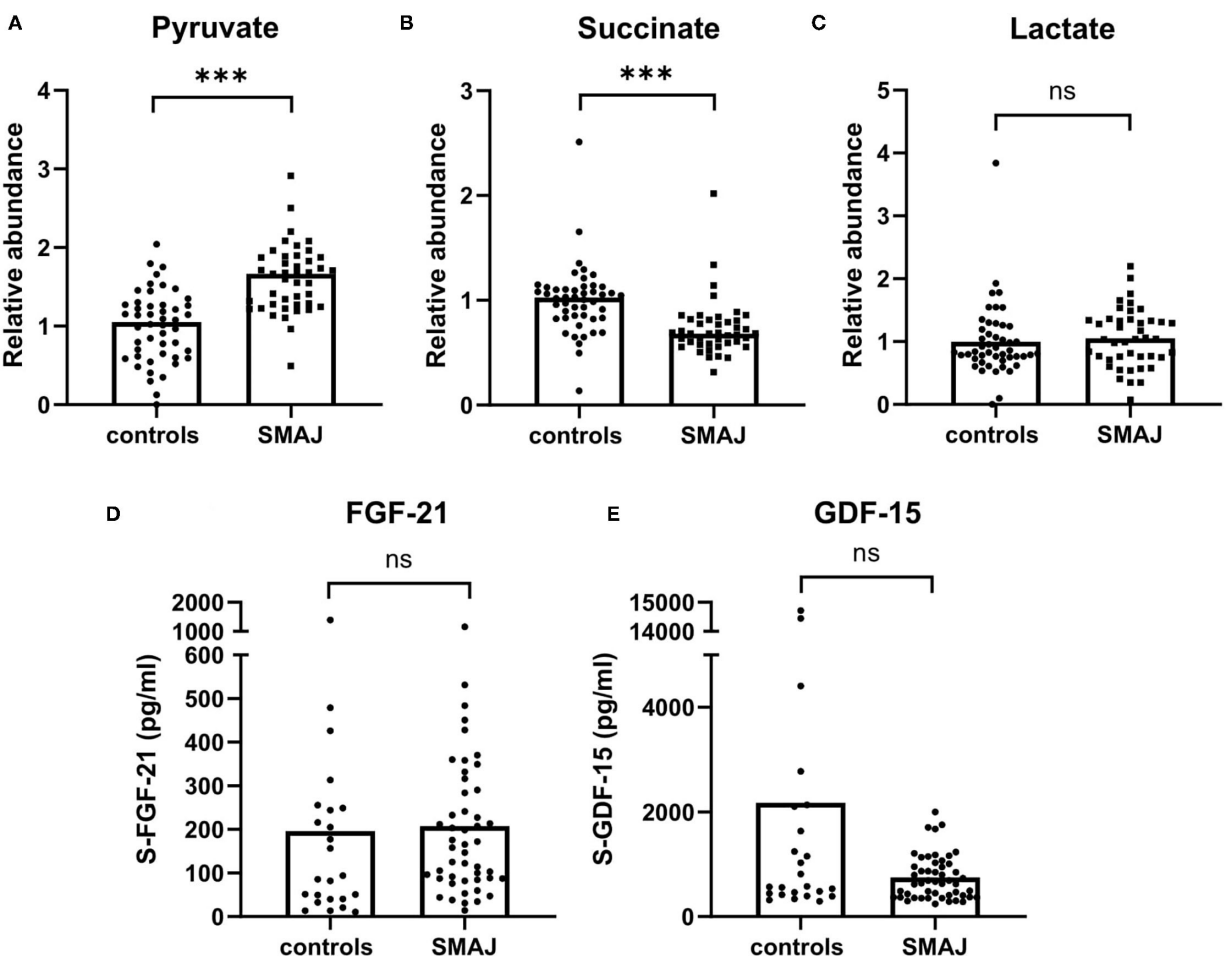
Mitochondrial disease biomarkers in SMAJ. Comparison of **(A)** pyruvate, **(B)** succinate, and **(C)** lactate values between patients with SMAJ (*n* = 43) and controls (*n* = 48) measured with a mass spectrometer. Comparison of **(D)** FGF-21 and **(E)** GDF-15 levels between patients with SMAJ patients (*n* = 49) and controls (*n* = 24) measured by ELISA assays. Median per group is indicated by a bar. ***p < 0.0001, Mann–Whitney *U*-test.

Metabolite profiling showed elevated levels of pyruvate (Figure 3A), whereas succinate was decreased in SMAJ (Figure 3B), indicating alterations related to mitochondrial metabolism. Lactate, however, was not different between the two groups (*p* = 0.34) (Figure 3C). These findings suggested that the mitochondrial origin of SMAJ contributed to the serum metabolite profile in patients. However, we did not find the level of mitochondrial ISR markers, FGF-21 or GDF-15 (22, 23) to differ between patients and control groups (FGF-21 SMAJ median, 162 pg/ml; controls, 90 pg/ml, [*p* = 0.15]; GDF-15 SMAJ median, 653 pg/ml; and controls, 574 pg/ml, [*p* = 0.29] (Figures 3D,E).

## Discussion

Blood biomarkers give information on systemic consequences, tissue involvement, and pathogenesis of a given disease or gene mutation. Moreover, they can serve as outcome measures for rare neurological diseases. Here, we characterized blood metabolites and biomarkers in a unique homogeneous cohort of patients with SMAJ with an identical *CHCHD10* mutation. Our main findings were (1) unlike in several other motor neuron diseases, the neurodegeneration biomarker, NfL, is not elevated in SMAJ. (2) Creatine metabolism is altered and could provide a biomarker for SMAJ, and (3) abnormal CHCHD10 has a mild effect on mitochondrial metabolism in SMAJ but does not induce ISR biomarkers.

The absence of elevation of NfL, despite a chronic injury process of spinal motor neurons in SMAJ (1), contrasts with high levels of neurofilaments, both NfL and pNFH (phosphorylated neurofilament heavy), in blood and CSF of patients with ALS (24, 25), and in children with recently diagnosed 5q-SMA (26, 27). However, adults and adolescents with 5q-SMA do not have a consistent NfL increase in CSF (28–30). The fact that SMAJ is slowly progressive and largely restricted to lower limbs may mean that the number of actively degenerating spinal motor neurons is not sufficient to elevate NfL. Nonetheless, this hypothesis is not supported by the elevation of serum NfL in patients with similarly slowly progressive inherited peripheral neuropathies (31). The absence of GFAP increase in SMAJ serum suggests that astrocytic injury or activation is not an important part of the disease process; the significantly lower levels of GFAP in patients with SMAJ compared with controls may suggest impaired astrocytic activation, but more studies are needed before any strong conclusions regarding this can be drawn. It is noteworthy that an autopsied individual did not have gliosis of the spinal cord (4).

Biomarker profiles of patients with SMAJ showed some similarities to patients with spinobulbar muscular atrophy (SBMA, MIM #313200), where muscle atrophy is, likewise, gradually progressive, that includes bulbar involvement. Patients with SBMA had normal blood NfL, but CK and creatine were increased, and creatinine was decreased (32, 33). The degree of muscle injury in SMAJ and SBMA appears enough to elevate the CK, which is in line with myopathic changes on the muscle biopsy being more prominent in these diseases than in ALS. Some degree of primary muscle pathology cannot be excluded in these diseases (3). In addition to creatinine, decreased levels of taurine and N-acetyl-L-carnosine may also be indicators of a decreased muscle mass in SMAJ. The CK did not correlate with the clinical severity in SBMA or ALS (32), but creatinine predicted the severity in both SBMA and SMA, particularly the Types 1 and 2 of the latter (32, 34). Creatinine also correlated with a therapeutic outcome of Nusinersen in SMA (35).

Serum lactate, which is a nonspecific marker for many mitochondrial diseases, particularly in children, was not increased in patients with SMAJ (36). However, we found elevated pyruvate in SMAJ. Altered pyruvate has been previously linked to primary mitochondrial myopathies (37, 38). Increased pyruvate, together with decreased succinate, suggests a block in the TCA cycle, and, thus, glycolysis may be upregulated. The altered metabolites may thus be directly involved in compensatory pathways for balancing an energy deficiency in SMAJ. Interestingly, a recent study of ALS-linked *CHCHD10* variant has reported similar findings for pyruvate and succinate in a patient's fibroblasts (14). The same study, as well as Chchd10 mouse models, has indicated an upregulation of ISR as part of pathogenesis, which leads to an increased expression of muscle-secreted FGF-21 and GDF-15 (8, 14). Nevertheless, here, we have shown that, in the blood of patients with CHCHD10-linked disease, these growth factors are not induced. It is, however, possible that since p.G66V produces a milder phenotype than the other *CHCHD10* mutations, it does not initiate the ISR.

As a limitation of this study, symptom severity could only be assessed retrospectively. To determine clinically useful biomarkers of SMAJ, it is necessary to perform longitudinal studies with validated measures of disease severity. Furthermore, although a large proportion of all patients with SMAJ was included in the study, the number of analyzed samples was still relatively small for a biomarker study. Further studies may also benefit from a larger control group, e.g., in a 1:2 ratio between patients and controls. The hypotheses generated here serve as a basis for further studies.

In conclusion, our study has established new insights into the pathophysiology of SMAJ in humans and identified metabolites, which may be assessed as clinical biomarkers in the future. Normal serum NfL may help differentiate SMAJ from early-stage ALS, and, perhaps, more generally, between rapidly and slowly progressive motor neuron diseases. Our findings on skeletal muscle-derived markers suggest that therapies designed for primary mitochondrial diseases may also be worth testing in SMAJ.

## Data Availability Statement

The raw data supporting the conclusions of this article will be made available by the authors, without undue reservation.

## Ethics Statement

This study was approved by the Medical Ethics Committee of Helsinki University Hospital. The patients/participants provided their written informed consent to participate in this study.

## Author Contributions

The study concept was contributed by JJ, SH, EP, MA, MJ, EY, and HT. Data collection was contributed by JJ. Analysis and interpretation of data were contributed by JJ, SH, EY, and HT. Statistical analysis was contributed by JKv. NfL and GFAP measurements were contributed by HZ. Metabolite profiling was contributed by JKo and ML. Patient data collection was contributed by AS and MJ. Drafting of the manuscript was contributed by JJ, SH, EY, and HT. Critical revision of the manuscript was contributed by all the authors.

## Funding

This study was supported by the Academy of Finland, University of Helsinki, Emil Aaltonen Foundation, Sigrid Juselius Foundation, Neurocenter Finland, and HUS Helsinki University Hospital VTR funds. HZ is a Wallenberg scholar supported by grants from the Swedish Research Council (#2018-02532), the European Research Council (#681712), Swedish State Support for Clinical Research (#ALFGBG-720931), the Alzheimer Drug Discovery Foundation (ADDF), USA (#201809-2016862), the AD Strategic Fund and the Alzheimer's Association (#ADSF-21-831376-C, #ADSF-21-831381-C, and #ADSF-21-831377-C), the Olav Thon Foundation, the Erling-Persson Family Foundation, Stiftelsen för Gamla Tjänarinnor, Hjärnfonden, Sweden (#FO2019-0228), the European Union's Horizon 2020 research and innovation program under the Marie Skłodowska-Curie grant agreement No. 860197 (MIRIADE) and the UK Dementia Research Institute at UCL.

## Conflict of Interest

HZ has served on Scientific Advisory Boards for Denali, Roche Diagnostics, Wave, Samumed, Siemens Healthineers, Pinteon Therapeutics, Nervgen and CogRx, has given lectures in symposia sponsored by Fujirebio, Alzecure and Biogen, and is a co-founder of Brain Biomarker Solutions in Gothenburg AB (BBS), which is a part of the GU Ventures Incubator Program. EY has served on Scientific Advisory Boards for Biogen Inc. and has received presentation fees from Roche Inc. and Sanofi Inc. The remaining authors declare that the research was conducted in the absence of any commercial or financial relationships that could be construed as a potential conflict of interest.

## Publisher's Note

All claims expressed in this article are solely those of the authors and do not necessarily represent those of their affiliated organizations, or those of the publisher, the editors and the reviewers. Any product that may be evaluated in this article, or claim that may be made by its manufacturer, is not guaranteed or endorsed by the publisher.
