# Supplementary material for: Serum Creatine, Not Neurofilament Light, Is Elevated in CHCHD10-Linked Spinal Muscular Atrophy
Source: Front Neurol. 2022 Feb 17;13:793937. doi: 10.3389/fneur.2022.793937 (PMC8891230; doi:10.3389/fneur.2022.793937)
Supplement: Supplementary file 1 [file Data_Sheet_1.docx]

**Supplementary material**

Supplementary Table 1. Symptom score for SMAJ patients, modified from the motor and sensory defect part of a more comprehensive CMT-score (15). In this study, certain patients’ symptoms were not strong enough to merit positive scores on this scale (e.g. only cramps), and thus received a symptom score of 0. Sufficient patient records were available for assessing the score for 43 patients. As SMAJ is a progressive disease, the symptom score correlated with age (r=0.56, p<0.001).

| Parameter | 0 | 1 | 2 | 3 | 4 |
| --- | --- | --- | --- | --- | --- |
| Sensory symptoms | None | Symptoms below or at ankle bones | Symptoms up to the distal half of the calf | Symptoms up to the proximal half of the calf, including knee | Symptoms above the knee (above the top of the patella) |
| Motor symptoms (legs) | None | Trips, catches toes, slaps foot, shoe inserts | Ankle support or stabilization (AFOs), foot surgery | Walking aids (cane, walker) | Wheelchair |
| Motor symptoms (arms) | None | Mild difficulty with buttons | Severe difficulty or unable to do buttons | Unable to cut most foods | Proximal weakness, (affect movements involving the elbow and above) |

Supplementary Table 2. Correlations between different markers. Adjusted p value < 0.05 was considered significant.

|  | Age | Sex | Symptom score | Creatine | Creatinine | CK | Taurine | N-acetyl-L-carnosine | Pyruvate | Succinate | FGF-21 | GDF-15 | GFAP | NfL |
| --- | --- | --- | --- | --- | --- | --- | --- | --- | --- | --- | --- | --- | --- | --- |
| Creatine | **r = 0.40** | p = 0.056 | **r = 0.43** |  | r = -0.16 | **r = 0.34** | r = 0.27 | r = - 0.25 | r = 0.038 | r = 0.17 | **r = 0.32** | r = 0.10 | **r = 0.41** | r = 0.27 |
|  | **p = 0.009** |  | **p = 0.009** |  | p = 0.31 | **p = 0.03** | p = 0.08 | p = 0.11 | p = 0.81 | p = 0.27 | **p = 0.04** | p = 0.52 | **p = 0.008** | p = 0.09 |
| Creatinine | r = 0.22 | p = 0.12 | r = - 0.11 | r = -0.16 |  | r = 0.15 | r = 0.015 | **r = 0.60** | r = 0.18 | r = 0.0078 | r = 0.0088 | r = 0.014 | r = 0.047 | r = 0.10 |
|  | p = 0.16 |  | p = 0.52 | p = 0.31 |  | p = 0.36 | p = 0.92 | **p < 0.001** | p = 0.24 | p = 0.96 | p = 0.96 | p = 0.93 | p = 0.77 | p = 0.53 |
| CK | r = 0.079 | **p < 0.0001** | r = 0.16 | **r = 0.34** | r = 0.15 |  | r = -0-0014 | r = 0.022 | r = - 0.10 | r = 0.28 | r = -0.14 | r = - 0.10 | r = - 0.18 | r = - 0.12 |
|  | p = 0.62 |  | p = 0.35 | **p = 0.03** | p = 0.36 |  | p > 0.99 | p = 0.89 | p = 0.52 | p = 0.07 | p = 0.34 | p = 0.48 | p = 0.21 | p = 0.43 |
| Taurine | r = 0.15 | p = 0.17 | r = -0.33 | r = 0.27 | r = 0.015 | r = -0-0014 |  | r = -0.019 | r = -0.19 | **r = 0.37** | r = 0.27 | r = -0.0094 | r = 0.22 | r = 0.30 |
|  | p = 0.32 |  | p = 0.05 | p = 0.08 | p = 0.92 | p > 0.99 |  | p = 0.90 | p = 0.23 | **p = 0.01** | p = 0.09 | p = 0.95 | p = 0.17 | p = 0.06 |
| N-acetyl-L-carnosine | r = - 0.032 | p = 0.35 | r = - 0.10 | r = - 0.25 | **r = 0.60** | r = 0.022 | r = -0.019 |  | r = 0.084 | r = 0.066 | r = - 0.083 | r = 0.21 | r = - 0.050 | r = -0.12 |
|  | p = 0.77 |  | p = 0.54 | p = 0.11 | **p < 0.001** | p = 0.89 | p = 0.90 |  | p = 0.59 | p = 0.67 | p = 0.61 | p = 0.18 | p = 0.75 | p = 0.44 |
| Pyruvate | r = 0.24 | p = 0.07 | r = 0.28 | r = 0.038 | r = 0.18 | r = - 0.10 | r = -0.19 | r = 0.084 |  | r = 0.082 | r = 0.054 | r = 0.072 | r = 0.14 | r = 0.018 |
|  | p = 0.13 |  | p = 0.10 | p = 0.81 | p = 0.24 | p = 0.52 | p = 0.23 | p = 0.59 |  | p = 0.60 | p = 0.74 | p = 0.65 | p = 0.37 | p = 0.91 |
| Succinate | r = 0.22 | p = 0.86 | r = - 0.044 | r = 0.17 | r = 0.0078 | r = 0.28 | **r = 0.37** | r = 0.066 | r = 0.082 |  | r = 0.11 | r = 0.17 | r = 0.046 | r = 0.25 |
|  | p = 0.16 |  | p = 0.80 | p = 0.27 | p = 0.96 | p = 0.07 | **p = 0.01** | p = 0.67 | p = 0.60 |  | p = 0.50 | p = 0.28 | p = 0.77 | p = 0.11 |
| FGF-21 | r = 0.022 | p = 0.96 | r = 0.043 | **r = 0.32** | r = 0.0088 | r = -0.14 | r = 0.27 | r = - 0.083 | r = 0.054 | r = 0.11 |  | **r = 0.35** | **r = 0.32** | r = 0.085 |
|  | p = 0.88 |  | p = 0.79 | **p = 0.04** | p = 0.96 | p = 0.34 | p = 0.09 | p = 0.61 | p = 0.74 | p = 0.50 |  | **p = 0.02** | **p = 0.03** | p = 0.57 |
| GDF-15 | r = 0.18 | p = 0.22 | r = 0.18 | r = 0.10 | r = 0.014 | r = - 0.10 | r = -0.0094 | r = 0.21 | r = 0.072 | r = 0.17 | **r = 0.35** |  | r = 0.25 | r = 0.084 |
|  | p = 0.21 |  | p = 0.26 | p = 0.52 | p = 0.93 | p = 0.48 | p = 0.95 | p = 0.18 | p = 0.65 | p = 0.28 | **p = 0.02** |  | p = 0.08 | p = 0.56 |
| GFAP | **r = 0.57** | p = 0.88 | r = 0.25 | **r = 0.41** | r = 0.047 | r = - 0.18 | r = 0.22 | r = - 0.050 | r = 0.14 | r = 0.046 | **r = 0.32** | r = 0.25 |  | **r = 0.59** |
|  | **p < 0.001** |  | p = 0.11 | **p = 0.008** | p = 0.77 | p = 0.21 | p = 0.17 | p = 0.75 | p = 0.37 | p = 0.77 | **p = 0.03** | p = 0.08 |  | **p < 0.001** |
| NfL | **r = 0.55** | p = 0.37 | r = 0.066 | r = 0.27 | r = 0.10 | r = - 0.12 | r = 0.30 | r = -0.12 | r = 0.018 | r = 0.25 | r = 0.085 | r = 0.084 | **r = 0.59** |  |
|  | **p < 0.001** |  | p = 0.68 | p = 0.09 | p = 0.53 | p = 0.43 | p = 0.06 | p = 0.44 | p = 0.91 | p = 0.11 | p = 0.57 | p = 0.56 | **p < 0.001** |  |
