# Supplementary figures and images for: Serum Creatine, Not Neurofilament Light, Is Elevated in CHCHD10-Linked Spinal Muscular Atrophy
Source: Front Neurol. 2022 Feb 17;13:793937. doi: 10.3389/fneur.2022.793937 (PMC8891230; doi:10.3389/fneur.2022.793937)

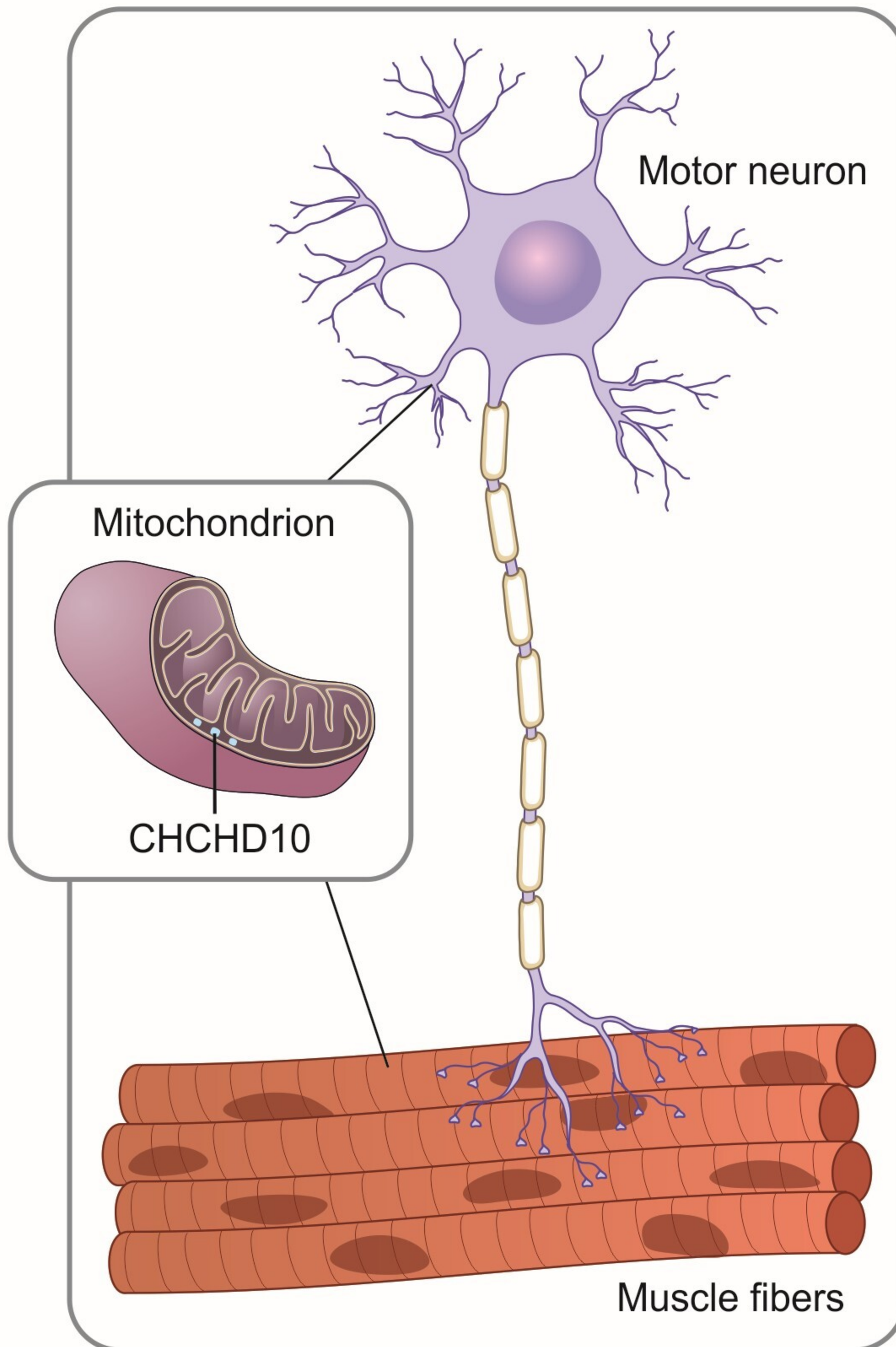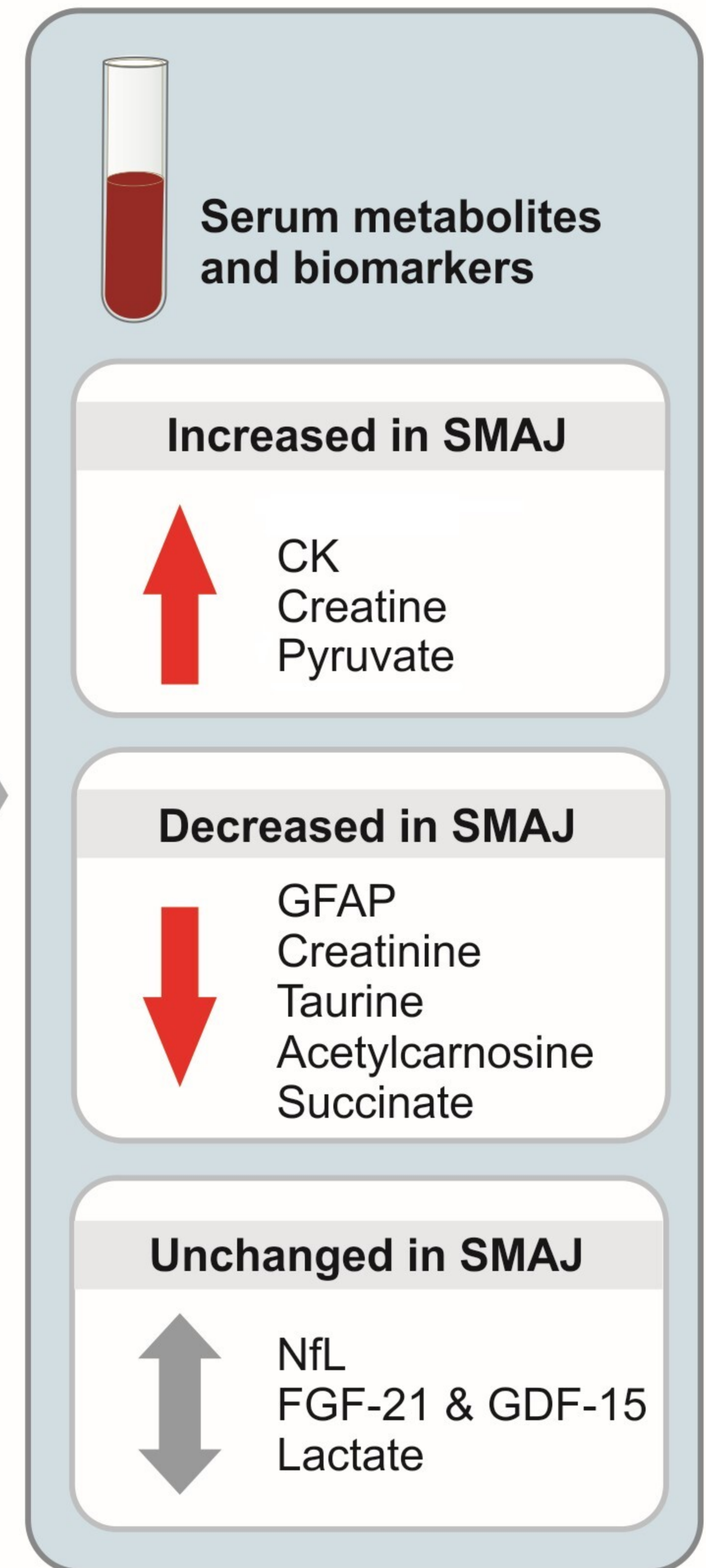

Supplement: Supplementary file 2 [file Image_1.pdf]
